# Relationship among methadone dose, polymorphisms of dopamine D2 receptor and tri-dimensional personality questionnaire in heroin-addicted patients

**DOI:** 10.1186/s12993-016-0109-9

**Published:** 2016-08-31

**Authors:** Min-Wei Huang, Tai-An Chiang, Pei-Yu Lo, Ching-Shan Huang

**Affiliations:** 1 Department of Psychiatry, Chia-Yi Branch, Taichung Veterans General Hospital, Chiayi, Taiwan; 2 General Education Center, Taiwan Shoufu University, Tainan, Taiwan; 3 College of Medicine and Life Science, Chung Hwa University of Medical Technology, Tainan, Taiwan; 4 Institute of Biomedical Engineering, National Cheng Kung University, Tainan, Taiwan; 5 Administration Center of Research and Education Innovation, Changhua Christian Hospital, Changhua, Taiwan

**Keywords:** *DRD2*, TPQ, Borderline index, Methadone requirement, Heroin-dependent patients

## Abstract

**Background:**

We investigated whether variation in the dopamine D2 receptor gene (*DRD2*) and tri-dimensional personality questionnaire (TPQ) scores could be used to aid adjustment of daily methadone requirements of heroin addicts. *DRD2* TaqI B polymorphisms and TPQ scores were determined in 138 male Taiwanese heroin addicts who were receiving methadone treatment. Borderline index (harm avoidance + novelty seeking—reward dependence) was calculated for each subject, and three groups were defined: high (mean from all subjects plus 1 standard deviation, or greater), low (half of the calculated high score, or lower) and medium (all values between the high and low scores).

**Results:**

No significant differences in age (*p* = 0.60), mean methadone dose (*p* = 0.75) or borderline index group (*p* = 0.25) were observed between subjects bearing the B1/B1, B1/B2 and B2/B2 *DRD2* TaqI genotypes. Among the individuals with low (≤10), medium (11–20) and high (≥21) borderline index scores, there was a significant difference in mean methadone dose (*p* = 0.04), but not age (*p* = 0.90). Further analysis showed that mean methadone dose was significantly higher in subjects with low borderline index scores than in those with high scores (62.5 vs. 47.0 mg/day, *p* = 0.03). The odds ratio for a daily methadone requirement ≥60 mg (median dose across the 138 subjects) was 2.64-fold greater in the low borderline index group than in the high group (*p* = 0.04).

**Conclusions:**

Although the *DRD2* TaqI B genotype was not associated with methadone use requirements, borderline index was revealed as a potential predictive marker for the adjustment of methadone dosage requirements in heroin addicts.

## Background

Rates of illicit opioid use are continuing to rise on a global scale, with North America being among the regions with most problematic levels of opioid use [1–3]. In Taiwan, heroin addiction is still a serious problem with approximately 50–100,000 people dependent on the drug, accounting for more than 90 % of all illicit drug users [4]. The situation has a dangerous impact on national health. This trend highlights the urgent need to focus on preventive measures against HCV and HIV infection caused by needle sharing among the heroin-using population. In order to reduce the occurrence of these public health threats, methadone, the cost-effective replacement for heroin dependence, was introduced as a maintenance treatment for heroin addiction in Taiwan in 2006 [4]. Since then, new HIV cases related to drug use by injection have declined steadily: 38.54 % in 2007, 35.87 % in 2008, 34.44 % in 2009, 32.15 % in 2010, 29.85 % in 2011 and 27.58 % in 2012 [4]. From the viewpoint of infection control, methadone treatment for heroin addiction is worthy of support. However, the quality of methadone treatment programs is a concern. Identifying the required methadone dose in each individual is crucial to successfully blocking the effects of heroin, thus reducing drug cravings and preventing relapses and adverse reactions [5].

Genetic factors that determine drug absorption, distribution, metabolism and action contribute to the large inter-individual variability in drug response [6]. Dopamine receptors play a major role in the rewarding effects of drugs of abuse [7]. Variations in the dopamine D2 receptor gene (*DRD2*) are strongly associated with opiate addiction [7]. Polymorphisms of *DRD2,* located in intron 2 (TaqI B), are significantly associated with polysubstance abuse in white, but not black, Americans [7]. Personality traits have been considerable heritable components and dopaminergic transmission has been suggested to influence certain personality traits, among them novelty seeking [8, 9]. The dopamine D2 receptor and its gene (*DRD2*) have also been associated with novelty seeking and related traits [10, 11]. Inherited behavioural propensity (temperament) is important for identifying substance-dependent patients and is strongly linked with the risk of drug abuse [11–14]. Cloninger has proposed a biosocial theoretical model of human personality based on three heritable independent dimensions: (1) Novelty *Seeking,* which is the tendency to respond actively to novel stimuli leading to pursuit of rewards and escape from punishment; (2) *Harm Avoidance,* which is the tendency toward an inhibitory response to signals of aversive stimuli that lead to avoidance of punishment and nonreward; and (3) *Reward Dependence,* which is the tendency for a positive response to signals of reward to maintain or resist behavioral extinction [9, 15–17]. Further developments in the model have preserved the three dimensions within a more comprehensive model of personality [18].

We hypothesised that *DRD2* gene variant expression and tri-dimensional personality questionnaire scores could be used to assist with adjustment of daily methadone requirements of heroin addicts. We therefore investigated, for the first time, *DRD2* TaqI B polymorphisms, tri-dimensional personality questionnaires (TPQs) and methadone dose requirements simultaneously in subjects addicted to heroin who were receiving methadone treatment.

## Methods

### Participants and procedures

We enrolled male Taiwanese heroin-dependent patients, aged 20 years or older and receiving methadone replacement therapy at the Chia-Yi Branch of Taichung Veterans General Hospital, Taiwan. All participants were unrelated, and born and living in Taiwan. Potential subjects with a history of HIV infection, severe hepatitis, severe structural heart disease or arrhythmias were excluded. A total of 138 subjects participated in the study. Written informed consent was obtained from each participant prior to enrolment, and the study was approved by the review board of the Chia-Yi Branch of Taichung Veterans General Hospital and the ethics board of the Central Taiwan University of Science and Technology.

Methadone was selected by the investigator to meet each patient’s requirements, in accordance with national guidelines, and was administered once daily. According to the clinical picture and therapy success, the dose of methadone was then gradually adapted to reach the maximum dose needed by that patient. Furthermore, a questionnaire was used to register the patients’ specific demographic characteristics (e.g., educations, marriage, whether use other substance or not, the manners and duration of using heroine, and dosage of heroine). Physical symptoms were measured using the opiate withdrawal scale. Urine screening tests were carried out regularly, but at random time intervals, to detect additional consumption. Urine samples of each client were always temperature tested. Patients with positive urine toxicology results were not enrolled in this study [19]. For all 138 study subjects, daily methadone doses were recorded during the predefined observation period of 15 months. The TPQ, a lOO-question self-report instrument, was developed to measure these dimensions and sub-dimensions [9, 15]. The TPQ was applied to survey all the 138 study subjects.

### Determination of *DRD2* TaqI B polymorphisms

Total genomic DNA was isolated from whole blood (2 ml) with EDTA as the anticoagulant, using a blood DNA isolation kit (Maxim Biotech Inc., San Francisco, CA, USA). DRD2 TaqI B expression was determined by PCR-restriction fragment length polymorphism as reported previously [20]. In brief, forward (5′-GATACCCACTTCAGGAAGTC-3′) and reverse (5′-GATGTGTAGGAATTAGCCAGG-3′) primers were used to amplify a 459 bp fragment. The reaction was carried out in a DNA thermal cycler (Applied Biosystems, Norwalk, CT, USA) as follows: denaturation at 94 °C for 4 min, 35 cycles of maintenance at 94 °C for 30 s, annealing at 58 °C for 30 s, and primer extension at 72 °C for 30 s; and a final extension step at 72 °C for 5 min. The PCR products were digested with Taq I, and analyzed on 3 % agarose gel (NuSieve 3:1, FMC Bioproduct, Rockland, ME, USA) containing ethidium bromide. One (459 bp), three (459, 267 and 192 bp) and two (267 and 192 bp) fragments were seen for B1/B1, B1/B2 and B2/B2 genotypes, respectively. Subjects were divided into three groups according to their *DRD2* TaqI B polymorphisms. The Taq1B polymorphism would be referred to by the number rs1079597 and the alleles referred to as A/G or T/C depending on the strand the genotype assay targets [21, 22].

### TPQ and borderline index assessments

The TPQ is a 100-item, self-administered, true/false instrument comprising three high-grade dimensions: harm avoidance, novelty seeking and reward dependence, each with four subscales. A three factor solution was extracted from analysis at the scale level which gives support to Cloninger’s model [23]. It is designed to measure biological component of personality. The assessment was conducted by two specialists in the Taiwanese version of the method [24, 26]. The Chinese version used in our study was developed by Chen et al. [23]. According to the normative data for TPQ in Taiwan, we divided our sample to eight personality patterns (for example, the norm of NS/HA/RD in Taiwan is 13.2/13.8/13.5, a profile with a NS/HA/RD of 18/17/10 is coded as ++−) [24]. Cloninger’s temperament cube theory assumes that each pattern is associated with a personality category [17, 26]. In the presented study, the borderline pattern (BP) and other patterns (OP) were separated into two groups in order to examine our hypothesis. To quantitatively calculate the borderline features into a single value, we applied the concept of a ‘‘borderline index’’ (BI) developed from Huang et al. [25]. BI is calculated from NS total + HA total − RD total = BI score, because high NS, high HA, low RD is the main feature of borderline personality in Cloninger’s temperament cube theory. Borderline index, defined as harm avoidance + novelty seeking—reward dependence, as reported recently in Taiwanese subjects [25], was calculated for each subject. The participants were then divided into three groups, based on their score; high (mean borderline index of the 138 study subjects plus 1 standard deviation (SD), or greater), low (half of the high score, or lower), and medium (scores between these high and low limits).

### Statistics

Mean age and mean daily methadone dose were compared across subjects in the three genotype groups, using ANOVA. Borderline index distribution across genotypes was compared using the Chi square test. ANOVA was also used to compare mean age and mean daily methadone dose among the three borderline index groups. The power analysis was performed to explore the effluence of sample size on the results; therefore the study enrolled 138 participants.

Statistical significance was defined as *p* < 0.05 and multiple comparisons were followed by Scheffe’s method for ANOVA. If significance was identified between several groups, the appropriate model was applied to calculate the odds ratio (OR) and 95 % confidence interval (CI) of the OR to evaluate risk for certain situations. All data were analyzed using SPSS version 18.0 for Windows (SPSS Inc., Chicago, IL, USA).

## Results

The mean (SD) age and mean daily methadone dose of the 138 patients were age 39.3 (7.9) years, education 11.3 (2.4) years and 56.8 (24.8) mg, respectively (Table 1). All the patients were treated at least for 15 months, and only heroin use. The harm avoidance, novelty seeking, reward dependence and borderline index were [mean (SD)] 15.2 (6.9), 18.1 (7.2), 20.2 (6.3) and 12.5 (8.2), respectively (Table 1). High borderline index was calculated to be 21 (12.5 + 8.2) and a low index was 10 [(12.5 + 8.2)/2]. Age, mean methadone dose and borderline index distribution did not differ significantly in subjects bearing B1/B1, B1/B2 and B2/B2 *DRD2* TaqI genotypes (*p* = 0.60, 0.75 and 0.25, respectively; Table 2). The frequency of the TaqI-B genotypes met criteria for Hardy–Weinberg equilibrium, p > 0.50, and are consistent with the frequencies reported in previous studies [27]. Across the three borderline index groups, a significant effect of mean methadone dose (*p* = 0.04), but not age (*p* = 0.90), was observed (Table 3). Further analysis showed that the mean methadone dose was significantly higher in subjects with a low (≤10) borderline index score than in those with a high (≥21) score (62.5 vs. 47.0 mg/day, *p* = 0.03), but no difference in mean dose was observed between medium and high (*p* = 0.12), and medium and low (*p* = 0.12) scoring subjects.

**Table 1:** Age, mean methadone daily requirement, TPQ scores and borderline index of the 138 study subjects

	Mean (SD)	Range
Age, year	39.3 (7.9)	21–57
Mean methadone dose, mg/day	56.8 (24.8)	3–140
Harm avoidance	15.2 (6.9)	0–31
Novelty seeking	18.1 (7.2)	0–34
Reward dependence	20.2 (6.3)	1–34
Borderline index	12.5 (8.2)	0–31

**Table 2:** Data among subjects with different *DRD2* TaqI polymorphisms

*DRD2* TaqI	Borderline index ≦10	Borderline index 11–20	Borderline index ≧21
B1/B1 N = 29	7	16	6
B1/B2 N = 56	23	22	11
B2/B2 N = 53	26	17	10
χ^2^	5.419		
*p* value	0.25

**Table 3:** Data among subjects with different borderline index

Borderline index	Age, year Mean (SD) [range]	Mean methadone dose, mg/day Mean (SD) [range]
≦10, N = 56	39.3 (6.8) [21–55]	62.5a (20.8) [15–95]
11–20, N = 55	39.5 (9.4) [26–57]	55.8b (26.6) [3–140]
≧21, N = 27	38.8 (7.2) [27–52]	47.0c (29.4) [5–90]
F	0.072	3.489
*p* value	0.90	0.04^a^

^a^ Multiple comparisons by Scheffe’s method show F = 1.945, *p* = 0.12 (a vs. b); F = 6.835, *p* = 0.03 (a vs. c) and F = 2.192, *p* = 0.12 (b vs. c), respectively

Therefore, borderline index score was considered a risk factor for daily methadone dose requirement. In a further analysis, a methadone dose of 60 mg per day (median dose among the 138 subjects) was used as the cut-off point and the OR for requiring a daily methadone dose ≥60 mg in subjects with high borderline index was defined as 1.0. In the borderline index assessment, the OR of requiring a methadone dose ≥60 mg/day was 2.64 times higher in subjects with a low score than in those with a high score (*p* = 0.04), whereas the OR (1.50) was not significant when medium- and high-scoring subjects were compared (*p* = 0.39) (Table 4).

**Table 4:** Odds ratios of methadone daily requirement ≥60 mg in subjects with different borderline index

	Methadone ≥60 mg/day	Methadone <60 mg/day	Odds ratio	95 % CI	*p* value
Borderline index					
≤10	38	18	2.64	1.03–6.78	0.04
11–20	30	25	1.50	0.59–3.79	0.39
≥21	12	15	1.0		

*CI* confidence interval

## Discussion

Methadone substitution or maintenance therapy is the most cost-effective and widely used treatment for heroin dependence. Optimal long-term outcomes require individualized dosing owing to large interindividual variability in methadone response [28].

The *DRD2* gene is located at chromosome 11q22–23 [29]. Genetic variants altering *DRD2* expression or function modulate the individual risk of opiate addiction, leading to the need for methadone substitution therapy [30–33]. For the *DRD2* gene, the polymorphism of most concern is TaqI A [34]. A1(+) allele carriers (A1/A1 and A1/A2 genotypes) have fewer brain D2 dopamine receptors than A1(−) allele carriers (A2/A2 genotype) [34]. It is thought that the TaqI A polymorphism renders the dopaminergic system inefficient and rewards substance abuse that increases brain dopamine levels [34]. However, in studies of methadone treatment for heroin addiction in white populations, the opposite association between the TaqI A polymorphism and methadone dose is observed [30–33]. TaqI B is found in high linkage disequilibrium with TaqI A [35]. We therefore investigated TaqI B polymorphisms for the subjects enrolled in the present study.

The TaqI B minor allele is significantly associated with 40 % fewer DRD2 receptor binding sites [36]. A significantly higher prevalence of the TaqI B1 allele is found in cocaine-dependent white subjects compared with non-substance-abusing controls [7]. Smokers homozygous for the TaqI B2 allele experience progressive improvement in self-reported withdrawal symptoms, whereas those with the TaqI-B1 allele show little change [37]. *DRD2* TaqI B is associated with alcoholism with conduct disorder in both white and Taiwanese subjects [38, 39]. However, our results revealed that TaqI B, similarly to TaqI A, does not play an important role in methadone dosage requirements, as reported in one study of white subjects [32]. In a recent Taiwanese study, subjects with the *DRD2* allele coding for −214A>G or 939C>T had a two-fold greater chance of requiring a lower methadone dose than non-carriers of the allele [40]. Recent studies have found a functional DRD2 polymorphism, which influences splicing of DRD2 and alters the ratio of short:long DRD2 in the brain. This polymorphism has also been associated with opioid and cocaine addiction, and is an interesting variant that warrants further study [41, 42]. *DRD2* is worthy of more comprehensive investigation among the Taiwanese population.

TPQ data have been reported in Taiwanese individuals carrying certain genetic polymorphisms, adolescents (including substance users and those with behavioural problems), healthy adults, and adults with alcoholism [24, 25, 39–57]. Those reports reveal personality–gene and personality–behaviour interactions. However, no relationship between TPQ and methadone pharmacology has been established to date. In a study of Yugoslavian opiate addicts, significantly greater novelty-seeking behaviour as well as significant divergences of harm avoidance and reward dependence were observed compared with the control group [13]. A recent study of Malaysians showed that heroin addicts had higher scores for novelty-seeking and harm-avoidance personality traits, but lower scores for reward dependence when compared with control subjects [15]. A recent study in Taiwanese heroin addicts reported that harm avoidance and novelty seeking scores were significantly higher in patients than in controls [57]. Conflicting results in Yugoslavians, Malaysians and the Taiwanese may be attributable to ethnic differences. However, therapeutic methadone dose was not addressed in the three previously cited studies [13, 15, 57]. In a recent study of heroin-dependent Taiwanese subjects, those with borderline personality [borderline index: 22.74 (SD 4.08), *n* = 22] showed greater sympathetic activity and less parasympathetic activity after taking methadone compared to other personalities [borderline index: 16.70 (4.36), *n* = 22] [25]. In that study, current methadone dose (around 38 mg/day) was not significantly different between the two groups, and the mean methadone dose was not investigated [25]. Therefore, to our knowledge, we are the first to study the relationship between mean therapeutic methadone dose and borderline score in heroin addicts. Our results show that the daily methadone dose is significantly higher among subjects with borderline index scores ≤10 than in those with scores ≥21. We also demonstrate that individuals with borderline index scores ≤10 had an approximately 2.6-fold greater chance of requiring daily methadone doses ≥60 mg than those with borderline index scores ≥21. This association between borderline index score and methadone dose is a novel finding.

The TPQ scores are associated with *DRD2* TaqI A polymorphisms in healthy American boys [58], Taiwanese subjects with anxiety, depression and alcohol dependence [47], methamphetamine-dependent Americans [59], Finns [60], Germans [61], Russians and Tatars [62] and heroin-dependent Malaysians [15], whereas non-association was observed in healthy French and Austrian subjects [63, 64] and in depressed patients in a New Zealand population [65]. The contradictory results among white, Malaysian and Taiwanese populations could be attributable to ethnic differences. In our study, we found no association between *DRD2* TaqI B polymorphisms and TPQ scores.

In addition to pharmacogenomics, cofactors such as age, pathology and sex are also important determinants of interindividual variability in drug efficacy. More information about the participants other than age should be considered in the future, such as the manners and duration of using heroine, and dosage of heroine et al. Maybe there are other factors influencing the dosage of methadone. In the present study, all the patients were men, and we excluded subjects with a history of HIV infection, severe hepatitis, severe structural heart disease or arrhythmias. Moreover, our results demonstrate that the difference in age between the groups was not significant. Therefore, our results have less interference from other cofactors than previous studies, leading to a more confident conclusion than those based only on pharmacogenomic factors. A limitation of this study is that the significant *p* values (0.03–0.04) obtained are only slightly smaller than 0.05; this may be attributable to the number of study subjects is not large enough. In this study, the further controlled design for population stratification should be considered to avoid confounding the association between genotype and the trait of interest.

Furthermore, our study does not investigate the mechanism underlying low borderline index scores predicting the need for higher methadone doses in heroin-dependent patients, and only TaqI B was determined for *DRD2* in our patients. Further investigation of *DRD2* variants in our patients is warranted. A more comprehensive study, with a larger sample size, investigating relationships between polymorphisms at nucleotides −214, 939, TaqI A and TaqI B in the *DRD2* gene, and TPQ scores, borderline index, sympathetic and parasympathetic activity, and methadone dose for treatment of heroin-dependent subjects, is ongoing in our laboratory.

## Conclusions

In conclusion, methadone use requirements in heroin-dependent subjects were associated with the borderline index score, but not with *DRD2* TaqI B genotype. Although the *DRD2* TaqI B genotype was not associated with methadone use requirements in this study, borderline index was revealed as a potential predictive marker for the adjustment of methadone dosage requirements in heroin addicts.

## Abbreviations

DRD2: dopamine D2 receptor gene
TPQ: tri-dimensional personality questionnaire
OWS: opiate withdrawal scale

## References

[1] Gomes T, Mamdani MM, Dhalla IA, Cornish S, Paterson JM, Juurlink DN (2014). The burden of premature opioid-related mortality. Addiction..

[2] United Nations Office on Drugs and Crime: World Drug Report. 2014. http://www.unodc.org/documents/wdr2014/World_Drug_Report_2014_web.pdf.

[3] Bawor Monica, Dennis Brittany B, Tan Charlie, Pare Guillaume, Varenbut Michael, Daiter Jeff, Plater Carolyn, Worster Andrew, Marsh David C, Steiner Meir, Anglin Rebecca, Desai Dipika, Thabane Lehana, Samaan Zainab (2015). Contribution of BDNF and DRD2 genetic polymorphisms to continued opioid use in patients receiving methadone treatment for opioid use disorder: an observational study. Addict Sci Clin Pract..

[4] Center for Disease Control Taiwan. National Statistics of Report. Center for Disease Control Taiwan, Taipei. 2013. http://www.cdc.gov.tw/En/.

[5] Epstein DH, Schmittner J, Umbricht A, Schroeder JR, Moolchan ET (2009). Promoting abstinence from cocaine and heroin with a methadone dose increase and a novel contingency. Drug Alcohol Depend.

[6] Kerb R (2006). Implications of genetic polymorphisms in drug transporters for pharmacotherapy. Cancer Lett.

[7] Kreek MJ, Bart G, Lilly C, LaForge KS, Nielsen DA (2005). Pharmacogenetics and human molecular genetics of opiate and cocaine addictions and their treatments. Pharmacol Rev.

[8] Loehlin JC. Genes and environment in personality development. Newbury Park: SAGE Publications; 1992.

[9] Cloninger CR (1986). A unified biosocial theory of personality and its role in the development of anxiety states. Psychiatr Dev.

[10] Suhara T, Yasuno F, Sudo Y, Yamamoto M, Inoue M, Okubo Y (2001). Dopamine D2 receptors in the insular cortex and the personality trait of novelty seeking. Neuroimage.

[11] Jönsson EG, Cichon S, Gustavsson JP, Grünhage F (2003). Association between a promoter dopamine D2 receptor gene variant and the personality trait detachment. Biol Psychiatr.

[12] Vukov M, Baba-Milkic N, Lecic D, Mijalkovic S, Marinkovic J (1995). Personality dimensions of opiate addicts. Acta Psychiatr Scand.

[13] Raby WN, Carpenter KM, Aharonovich E, Rubin E, Bisaga A (2006). Temperament characteristics, as assessed by the tridimensional personality questionnaire, moderate the response to sertraline in depressed opiate-dependent methadone patients. Drug Alcohol Depend.

[14] Teh LK, Izzuddin AF, Fazleen Haslinda MH, Zakaria ZA, Salleh MZ (2011). Tridimensional personalities and polymorphism of dopamine D2 receptor among heroin addicts. Biol Res Nurs.

[15] Cloninger CR (1987). A systematic method for clinical description and classification of personality variants. Arch Gen Psychiatr.

[16] Cioninger CR (1988). A unified biosocial theory of personality and its role in the development of anxiety states: a reply to commentaries. Psychiatr Develop.

[17] Cloninger CR, Svrakic DM, Przybeck TR (1993). A psychobiological model of temperament and character. Arch Gen Psychiatr.

[18] Strakowski SM, Dunayevich E, Keck PE, McElroy SL (1995). Affective state dependence of the tridimensional personality questionnaire. Psychiatr Res.

[19] Giacomuzzi SM, Ertl M, Kemmler G, Riemer Y, Vigl A (2005). Sublingual buprenorphine and methadone maintenance treatment: a three-year follow-up of quality of life assessment. Sci World J..

[20] Wu X, Hudmon KS, Detry MA, Chamberlain RM, Spitz MR (2000). D2 dopamine receptor gene polymorphisms among African-Americans and Mexican-Americans: a lung cancer case-control study. Cancer Epidemiol Biomarkers Prev.

[21] Levran O, Londono D, O‘Hara K, Nielsen DA, Peles E, Rotrosen J (2008). Genetic susceptibility to heroin addiction: a candidate gene association study. Genes Brain Behav.

[22] http://snpedia.com/index.php/Rs1079597.

[23] Stewart Mary E, Ebmeier Klaus P, Deary Ian J (2004). The structure of Cloninger’s tridimensional personality questionnaire in a British sample. Pers Individ Differ.

[24] Chen WJ, Chen HM, Chen CC, Chen CC, Yu WY (2002). Cloninger’s tridimensional personality questionnaire: psychometric properties and construct validity in Taiwanese adults. Compr Psychiatr.

[25] Huang WL, Lin YH, Kuo TB, Chang LR, Chen YZ (2012). Methadone-mediated autonomic functioning of male patients with heroin dependence: the influence of borderline personality pattern. PLoS ONE.

[26] Svrakic DM, Draganic S, Hill K, Bayon C, Przybeck TR (2002). Temperament, character, and personality disorders: etiologic, diagnostic, treatment issues. Acta Psychiatr Scand.

[27] Crttol Séverine, Besson Jacques, Croquette-Krokar Marina, Hämmig Robert (2008). Association of dopamine and opioid receptor genetic polymorphisms with response to methadone maintenance treatment. Prog Neuropsychopharmacol Biol Psychiatr.

[28] Li Y, Kantelip JP, Gerritsen-van Schieveen P, Davani S (2008). Interindividual variability of methadone response: impact of genetic polymorphism. Mol Diagn Ther..

[29] Grandy DK, Litt M, Allen L, Marchionni M, Makam H (1989). The human dopamine D2 receptor gene is located on chromosome 11 at q22–q23 and identifies a TaqI RFLP. Am J Hum Genet.

[30] Lawford BR, Young RM, Noble EP, Sargent J, Rowell J (2000). The D(2) dopamine receptor A(1) allele and opioid dependence: association with heroin use and response to methadone treatment. Am J Med Genet.

[31] Barratt DT, Coller JK, Somogyi AA (2006). Association between the DRD2 A1 allele and response to methadone and buprenorphine maintenance treatments. Am J Med Genet B Neuropsychiatr Genet.

[32] Crettol S, Besson J, Croquette-Krokar M, Hämmig R, Gothuey I (2008). Association of dopamine and opioid receptor genetic polymorphisms with response to methadone maintenance treatment. Prog Neuropsychopharmacol Biol Psychiatr.

[33] Doehring A, Hentig N, Graff J, Salamat S, Schmidt M (2009). Genetic variants altering dopamine D2 receptor expression or function modulate the risk of opiate addiction and the dosage requirements of methadone substitution. Pharmacogenet Gen.

[34] Noble EP (2000). Addiction and its reward process through polymorphisms of the D2 dopamine receptor gene: a review. Eur Psychiatr.

[35] Kidd KK, Morar B, Castiglione CM, Zhao H, Pakstis AJ (1998). A global survey of haplotype frequencies and linkage disequilibrium at the DRD2 locus. Hum Genet.

[36] Ritchie T, Noble EP (2003). Association of seven polymorphisms of the D2 dopamine receptor gene with brain receptor-binding characteristics. Neurochem Res.

[37] Robinson JD, Lam CY, Minnix JA, Wetter DW, Tomlinson GE (2007). The DRD2 TaqI-B polymorphism and its relationship to smoking abstinence and withdrawal symptoms. Pharmacogenomics J.

[38] Blum K, Noble EP, Sheridan PJ, Montgomery A, Ritchie T (1993). Genetic predisposition in alcoholism: association of the D2 dopamine receptor TaqI B1 RFLP with severe alcoholics. Alcohol.

[39] Lu RB, Lee JF, Ko HC, Lin WW (2001). Dopamine D2 receptor gene (DRD2) is associated with alcoholism with conduct disorder. Alcohol Clin Exp Res.

[40] Hung CC, Chiou MH, Huang BH, Hsieh YW, Hsieh TJ (2011). Impact of genetic polymorphisms in ABCB1, CYP2B6, OPRM1, ANKK1 and DRD2 genes on methadone therapy in Han Chinese patients. Pharmacogenomics.

[41] Moyer RA, Wang D, Papp AC, Smith RM, Duque L, Mash DC, Sadee W (2011). Intronic polymorphisms affecting alternative splicing of human dopamine D2 receptor are associated with cocaine abuse. Neuropsychopharmacology.

[42] Clarke TK, Weiss ARD, Ferraro TN, Kampman KM, Dackis CA, Pettinati HM, O’Brien CP, Oslin DW, Lohoff FW, Berrettini WH (2014). The dopamine receptor D2 (DRD2) SNP rs1076560 is associated with opioid addiction. Ann Hum Genet.

[43] Ko CH, Yen JY, Chen CC, Chen SH, Wu K (2006). Tridimensional personality of adolescents with internet addiction and substance use experience. Can J Psychiatr.

[44] Ko CH, Hsiao S, Liu GC, Yen JY, Yang MJ (2009). The characteristics of decision making, potential to take risks, and personality of college students with Internet addiction. Psychiatr Res.

[45] Kuo PH, Yang HJ, Soong WT, Chen WJ (2002). Substance use among adolescents in Taiwan: associated personality traits, incompetence, and behavioral/emotional problems. Drug Alcohol Depend.

[46] Kuo PH, Chih YC, Soong WT, Yang HJ, Chen WJ (2004). Assessing personality features and their relations with behavioral problems in adolescents: tridimensional personality questionnaire and junior Eysenck personality questionnaire. Compr Psychiatr.

[47] Lin SC, Wu PL, Ko HC, Wu JY, Huang SY (2007). Specific personality traits and dopamine, serotonin genes in anxiety-depressive alcoholism among Han Chinese in Taiwan. Prog Neuropsychopharmacol Biol Psychiatr.

[48] Tsai SJ, Wang YC, Hong CJ (2001). Allelic variants of the alpha1a adrenoceptor and the promoter region of the alpha2a adrenoceptor and temperament factors. Am J Med Genet.

[49] Tsai SJ, Wang YC, Hong CJ (2002). Norepinephrine transporter and alpha(2c) adrenoceptor allelic variants and personality factors. Am J Med Genet.

[50] Tsai SJ, Hong CJ, Cheng CY (2002). Serotonin transporter genetic polymorphisms and harm avoidance in the Chinese. Psychiatr Genet.

[51] Tsai SJ, Wang YC, Chen JY, Hong CJ (2003). Allelic variants of the tryptophan hydroxylase (A218C) and serotonin 1B receptor (A-161T) and personality traits. Neuropsychobiology.

[52] Tsai SJ, Yu YW, Hong CJ (2004). Personality traits in young female apolipoprotein E (apoE) epsilon4 and non-epsilon4 carriers. Am J Med Genet B Neuropsychiatr Genet.

[53] Tsai SJ, Hong CJ, Yu YW, Chen TJ (2004). Association study of catechol-O-methyltransferase gene and dopamine D4 receptor gene polymorphisms and personality traits in healthy young Chinese females. Neuropsychobiology.

[54] Wu CY, Wu YS, Lee JF, Huang SY, Yu L (2008). The association between DRD2/ANKK1, 5-HTTLPR gene, and specific personality trait on antisocial alcoholism among Han Chinese in Taiwan. Am J Med Genet B Neuropsychiatr Genet.

[55] Yu YW, Yang CW, Wu HC (2005). Association study of a functional MAOA-uVNTR gene polymorphism and personality traits in Chinese young females. Neuropsychobiology.

[56] Yeh YW, Lu RB, Tao PL, Shih MC, Lin WW (2010). Neither single-marker nor haplotype analyses support an association between the dopamine transporter gene and heroin dependence in Han Chinese. Genes Brain Behav.

[57] Yeh YW, Lu RB, Tao PL, Shih MC, Huang SY (2011). A possible association of the norepinephrine transporter gene in the development of heroin dependence in Han Chinese. Pharmacogenet Gen.

[58] Noble EP, Ozkaragoz TZ, Ritchie TL, Zhang X, Belin TR (1998). D2 and D4 dopamine receptor polymorphisms and personality. Am J Med Genet.

[59] Han DH, Yoon SJ, Sung YH, Lee YS, Kee BS (2008). A preliminary study: novelty seeking, frontal executive function, and dopamine receptor (D2) TaqI A gene polymorphism in patients with methamphetamine dependence. Compr Psychiatr.

[60] Keltikangas-Järvinen L, Pulkki-Råback L, Elovainio M, Raitakari OT, Viikari J (2009). DRD2 C32806T modifies the effect of child-rearing environment on adulthood novelty seeking. Am J Med Genet B Neuropsychiatr Genet.

[61] Montag C, Markett S, Basten U, Stelzel C, Fiebach C (2010). Epistasis of the DRD2/ANKK1 Taq Ia and the BDNF Val66Met polymorphism impacts novelty seeking and harm avoidance. Neuropsychopharmacology.

[62] Kazantseva A, Gaysina D, Malykh S, Khusnutdinova E (2011). The role of dopamine transporter (SLC6A3) and dopamine D2 receptor/ankyrin repeat and kinase domain containing 1 (DRD2/ANKK1) gene polymorphisms in personality traits. Prog Neuropsychopharmacol Biol Psychiatr.

[63] De Brettes B, Berlin I, Laurent C, Lépine J, Mallet J (1998). The dopamine D2 receptor gene TaqI A polymorphism is not associated with novelty seeking, harm avoidance and reward dependence in healthy subjects. Eur Psychiatr.

[64] Gebhardt C, Leisch F, Schüssler P, Fuchs K, Stompe T (2000). Non-association of dopamine D4 and D2 receptor genes with personality in healthy individuals. Psychiatr Genet.

[65] Light KJ, Joyce PR, Luty SE, Mulder RT, Carter JD (2007). An association study of DRD2 and COMT polymorphisms with novelty seeking and harm avoidance scores, in two independent samples of depressed patients. Behav Brain Funct.

